# Should Governments engage health insurance intermediaries? A comparison of benefits with and without insurance intermediary in a large tax funded community health insurance scheme in the Indian state of Andhra Pradesh

**DOI:** 10.1186/s12913-015-1028-4

**Published:** 2015-09-10

**Authors:** Srikant Nagulapalli, Sudarsana Rao Rokkam

**Affiliations:** Department of Economics, Andhra University, Karachettu Road, Visakhapatnam, India

## Abstract

**Background:**

A peculiar phenomenon of engaging insurance intermediaries for government funded health insurance schemes for the poor, not usually found globally, is gaining ground in India. Rajiv Aarogyasri Scheme launched in the Indian state of Andhra Pradesh, is first largest tax funded community health insurance scheme in the country covering more than 20 million poor families. Aarogyasri Health Care Trust (trust), the scheme administrator, transfers funds to hospitals through two routes one, directly and the other through an insurance intermediary. The objective of this paper is to find out if engaging an insurance intermediary has any effect on cost efficiency of the insurance scheme.

**Methods:**

We used payment data of RAS for the period 2007–12, to find out the influence of insurance intermediary on the two variables, benefit cost ratio defined as benefit payment divided by premium payment, and claim denial ratio defined as benefit payment divided by treatment cost. Relationship between scheme expenditure and number of beds empanelled under the scheme is examined. OLS regression is used to perform all analyses.

**Results:**

We found that adding an additional layer of insurance intermediary between the trust and hospitals reduced the benefit cost ratio under the scheme by 12.2 % (p-value = 0.06). Every addition of 100 beds under the scheme increases the scheme payments by US$ 0.75 million (p-value < 0.001). The gap in claim denial ratio between insurance and trust modes narrowed down from 2.84 % in government hospitals to 0.41 % in private hospitals (p-value < 0.001).

**Conclusions:**

The scheme is a classic case of Roemer's principle in operation. Introduction of insurance intermediary has the twin effects of reduction in benefit payments to beneficiaries, and chocking fund flow to government hospitals. The idea of engaging insurance intermediary should be abandoned.

## Background

In 2007, Andhra Pradesh introduced Rajiv Aarogyasri community health insurance Scheme (RAS) for treating the catastrophic illnesses of the 21 million Below Poverty Line (BPL) families requiring in-patient admissions. The scheme, implemented by Aarogyasri Health Care Trust (AHCT or trust), provides end-to-end cashless services for identified tertiary care diseases through a network of Government and private hospitals [1]. RAS is the first large scale demand side financed health insurance scheme launched in India. While on one hand, the scheme gave great political dividends due to the exceptional goodwill generated among the masses, on the other, it received a lot of criticism that a disproportionately large share of tax money is being routed to the private hospitals on a small number of tertiary care patients. In this paper, we study the fund transfer mechanism under the scheme in order to find out whether engaging an insurance intermediary has a positive or negative impact on the scheme.

The Government of Andhra Pradesh (GoAP) launched the scheme initially with around 300 treatments or therapies with the help of an insurance intermediary; the method of implementation being commonly referred as insurance mode. An year later in 2008, GoAP introduced about 600 additional treatments directly administered by trust; the implementation method commonly referred as trust mode. A total of 938 treatments were launched under the scheme in all the 23 districts and five regions of the state by 2008. The state was divided into five geographical regions for the sake of convenience while implementing the scheme and each region offered all the 938 therapies. In each region, AHCT offered some of the 938 therapies through insurance mode and some through trust mode. The mix of therapies under the insurance mode and the trust mode changed in each region every year. During the period 2008–2011, the number of therapies offered under insurance mode ranged between 192 and 938 while that under the trust mode ranged between 586 to 938 [2]. The insurance intermediary is selected through a bidding process. GoAP transfers the yearly RAS budget through periodical advances to the trust bank account. Trust transfers this amount under the head 'bulk premium' to the insurance intermediary as half-yearly advances. Hospitals willing to offer services under the scheme make an application to the Trust. The Trust inspects such hospitals and registers (empanels) them if they meet the required quality standards. Trust empanels only hospitals with more than 50 beds. An individual needing tertiary care, hailing from any region, is free to approach any hospital. The hospital makes a real-time preauthorisation request on the Information Technology (IT) platform of the trust, asking for permission to treat the case. Trust issues pre-authorisation to the case, and hospital treats the patient and submits a claim on the IT platform of the Trust. Depending on who is administering the type of therapy in that region at the time of preauthorisation, the insurance intermediary or the trust makes the payment to the hospital. By 2013, AHCT phased out the insurance intermediary and was administering all the 938 procedures through the trust mode. Against this backdrop, there exists an alternate view that engaging an insurance intermediary is necessary to run the scheme efficiently, control Supplier Induced Demand (SID), and reduce the costs. SID is the situation where a doctor, exploiting the information asymmetry, advises the patient to consume more care than is required clinically.

Our literature search did not yield any successful examples in the developed or developing countries in the world where countries transferred tax money directly to private insurance companies for administering wholly government funded health insurance schemes for their people. The advocates of insurance intermediary administered government funded health insurance schemes in India put forth the arguments of government inexperience, controlling SID, risk absorption, efficient operations, quality of care, controlling moral hazard and outsourcing of manpower as the reasons for engaging the insurance intermediary.

AHCT states that it did not have the expertise and the time to roll out a large scale health insurance scheme at short notice in April, 2007. Therefore, an insurance intermediary was engaged to help AHCT set up an implementation system and later run on its own [9]. Third Party Administrator (TPA) is a management contractor for health insurance firms in India, licenced by the Insurance Regulatory and Development Authority of India (IRDA). The experience of Vajpayee Aarogyasri of Karnataka state, launched after RAS and implemented without any insurance intermediary shows that the TPA route is a viable alternative. One hurdle to the TPA route is the prohibition by IRDA on TPAs to serve Government funded health insurance schemes directly. However, TPAs have been circumventing this prohibition by floating sister companies to service such schemes.

The Government created AHCT, an autonomous trust, as the single-payer health insurance agency to implement RAS. From 2013 onwards, AHCT has been successfully administering the scheme in the entire state through the trust mode without an insurance intermediary. The trust set up with tax money and answerable to the tax-payer, cannot express inability to perform the task assigned to it under its deed of formation [10].

There is a presumption that recruiting manpower permanently on the rolls of the government renders them lazy and useless because of the job security that a public job provides. Permanent recruitment in the state is restricted to the barest minimum and a policy of hiring through third party agencies is being adopted for the last twenty years. Almost a third of the government is manned by outsourced staff currently [11]. Hiring staff through third parties by government has seen many problems such as creation of hope of permanent absorption, nepotism during recruitment, non-payment of statutory allowances by the manpower hirers, suicides, strikes for grievance redressal and so on. The suicides and agitations by staff manning the government run ‘108’ ambulance services and other health services such as ‘104’ services, outsourced to some of the best private firms in the country, resulting in frequent disruption of emergency services across the country are evidence of the failure of growing reliance on outsourced staff [12–17]. It may not be possible for government to disown the responsibility of outsourced staff delivering cutting edge services by distancing itself from the management contractor who hires the staff. During elections voters question the government for delivery of services and not the management contractor. In the absence of a systematic government hiring policy and contract management, mere replacement of permanent manpower with outsourced staff is unlikely to yield desired results. A large field level workforce of more than 2000 staff continue to work for RAS, with or without an insurance intermediary, as long as the government policy continues the scheme. Once such a large trained workforce is under deployment, it is not possible to retrench them and recruit a fresh set of people every time a new insurance intermediary is engaged. The issues relating to staff persist even if a TPA is engaged because as long as the government policy supports demand side financing, the staff hired under the scheme cannot be retrenched.

Trust also made sustained efforts to improve the quality of service in both government and private hospitals with varying success. While government hospitals were slow to respond to quality standards the response of private hospitals is more encouraging. Furthermore, it is also widely recognised that the ration card is owned not only by BPL families but also APL families in the state. This strategy has helped move RAS closer to being a universal health care coverage scheme.

Starting with a budget of about US$10 million in 2007, RAS consumption stood stably at around US$200 million during the years 2012 and 2013. Officials of the trust state that the number of hospitals empanelled under RAS influence the total expenditure under the scheme. Roemer's law says that in an insured population, a hospital bed built is a filled bed; this forms the basis for the argument of SID in healthcare [3]. Trust officials state that Roemer's principle is often used by the insurance intermediary in regulating the hospitals empanelled under RAS in order to reduce the benefit payments to hospitals. The tenth recommendation of High Level Expert Group(HLEG) report on Universal Health Coverage(UHC) for India says that purchases of all health care services under the UHC system should be undertaken directly by government or through quasi-governmental autonomous agencies and not through insurance intermediaries [4]. In view of the conflict between HLEG recommendation and views expressed by certain quarters, this paper studies the experience of RAS in order to answer the question: should government engage an insurance intermediary for running Rajiv Aarogyasri Scheme?

Benefit Cost Ratio(BCR) generally indicates the efficiency with which expenditure of public funds is made under a health scheme. Similarly, the denial of payments to public hospitals and private hospitals would reveal discrimination if any. This study therefore assesses the impact of engaging an insurance intermediary on BCR and claim denials to hospitals by comparing the insurance and trust modes.

## Methods

### Study variables

The following variables were used in the analysis:

1. Claim Ratio or Benefit Cost Ratio(BCR) is defined as claim paid divided by premium paid. Claim paid is the benefit payments made to the hospitals either by the insurance intermediary or the trust. Premium for insurance mode is the amount paid by AHCT to the insurance intermediary inclusive of sales tax. Premium for the trust mode is calculated notionally as the sum of administrative cost plus claim paid. Administrative cost of insurance mode is calculated by deducting the claim paid from the premium paid.
2. Claim Denial Ratio (CDR) is defined as the amount denied divided by treatment cost. Treatment cost is the total cost of treatments done by the hospitals out of preauthorised treatments. Amount denied is the amount which remains undisbursed out of the treatment cost.
3. Hospitals is the number of empanelled hospitals existing as on 1st April of the year.
4. Beds is the total number of beds in all the empanelled hospitals as on 1st April of the year.
5. Payments is the amount disbursed to empanelled hospitals under the scheme.
6. Year refers to calendar year.
7. Mode refers to insurance mode or trust mode.
8. Hospital type is private hospital or government hospital.

### Data

The data relating to payments to hospitals, premium payments, administrative costs and empanelment of hospitals was obtained from AHCT for the period 2007-2012 [5, 2]. 1470(2.5 %) of the patients discharged during March and April, 2013 were sampled through simple random sampling and were interviewed by telephone from the State ‘104’ call centre in order to assess the patient satisfaction rates. ‘104’ is the name given to the 24x7 call centre run by the Government for providing health advise and information to the citizens. The respondents were asked from ‘104’ call centre if the treatment was very bad, bad, average, good or very good. Data for patient satisfaction rates was obtained from ‘104’ call centre [2].

### Data analysis

The analysis involved comparison of BCR and CDR with and without the insurance intermediary by using linear regression models. AHCT prescribed a standard preauthorisation and claims procedure for all the 938 therapies, the five regions of the state as well as both modes of implementation. Insurance intermediary as well as trust processed the preauthorisation and claims for all types of therapies and for all the five regions in the state on the same IT platform provided by AHCT. In view of this standardisation, the variables viz., region and type of therapy were omitted from the analysis. Data for patient satisfaction rates was available only without insurance intermediary; no comparison could therefore be made with and without insurance intermediary.

We used the following linear regression model for estimating the impact of insurance intermediary on BCR.

$$ \mathrm{B}\mathrm{C}\mathrm{R}=\alpha + {\beta}_1* mode $$

where, mode is a dummy variable equal to 0 for insurance mode and 1 for trust mode.

As AHCT did not administer any part of the scheme during the first year (2007), the administrative cost was imputed using Bayesian linear regression method [6].

The influence of hospital type on difference in CDR between the insurance and trust modes was examined using the model

$$ CD{R}_{insurance}-CD{R}_{trust}=\alpha + {\beta}_1* hospital\  type $$

where, hospital type is a dummy variable equal to 0 for government hospitals and 1 for private hospitals.

For testing the influence of hospital beds on scheme payments we used the linear regression model below.

$$ \mathrm{Payments}=\alpha + {\beta}_1* beds $$

Testing for heteroskedasticity and functional form was done using Breusch-Pagan test and Ramsey's RESET test. Robust testing using heteroskedasticity consistent procedure for small samples was done for all the three linear regression models [7].

### Ethics statement

The survey in this study was conducted with the approval of Government of Andhra Pradesh, India which also permitted publication of the survey results of the treated patients [8]. This research is in compliance with the Helsinki Declaration. AHCT which is a Government Trust obtained informed consent prior to the conduct of survey over telephone and all the participants were adults. AHCT Parents or guardians served as proxy for those below 18 years.

## Results and discussion

**Fig. 1:**
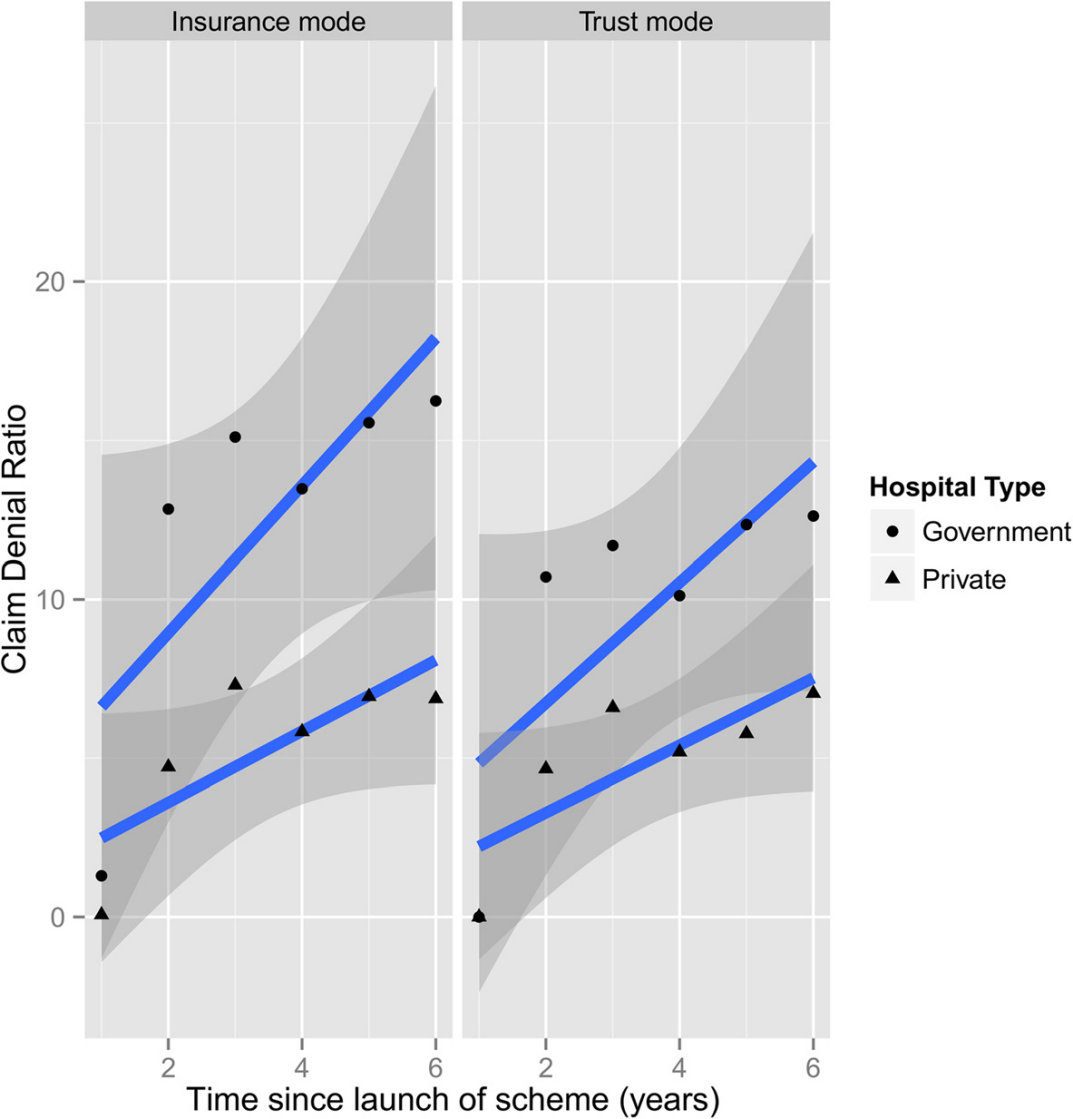
Claim Denial Ratio. Smoothed line by hospital type, faceted by implementation mode, with 95 % confidence intervals

Table 1 provides a summary of annual claim ratios during the period 2007 to 2012. The average claim ratio for insurance mode (81.4 %) was lower than that of trust mode (93.6 %). Table 2 presents the patient satisfaction rates estimated when the scheme was being fully administered under the trust mode. Regression results showed that adding an additional layer of insurance intermediary between the trust and hospitals reduced the benefit cost ratio under the scheme by 12.2 % (p-value = 0.06) (see Table 3). Table 4 presents the linear regression results of relationship between hospital benefit payments and empanelled hospital beds. We found that every addition of 100 beds under the scheme increases the scheme payments by US$ 0.75 million (p-value = 0). Table 5 gives the regression results of change of gap in CDR between insurance and trust modes. The gap in claim denial ratio between insurance and trust modes narrowed down from 2.84 % in government hospitals to 0.41 % in private hospitals (p-value = 0). Figure 1 shows that claim denials increased in government hospitals when payments were made through insurance intermediary.

**Table 1:** Claim ratio. Summary of claim ratio by implementation mode

	Min.	1st Qu.	Median	Mean	3rd Qu.	Max.
Insurance mode	70.9	72.3	81.6	81.4	85.9	96.3
Trust mode	84.6	88	96.5	93.62	96.6	102.4

**Table 2:** Patient satisfaction rate. Percentage of respondents expressing satisfaction when they were treated under trust mode

	Very bad	Bad	Average	Good	Very good	
Percentage	0.5	1.5	1.9	84.6	11.5	

Source-Telephonic survey conducted on 1470 discharged patients during March and April 2013 through 104 call centre outbound call services of AHCT

**Table 3:** Regression results of benefit cost ratio by implementation mode

	Dependent variable: Benfit-cost ratio	
	Naive	Robust
Trust mode	12.22* (1.11, 23.33)	12.22** (2.28, 22.15)
Constant	81.40*** (73.55, 89.25)	81.40*** (73.22, 89.58)
Observations	10	10
R square	0.37	0.37
Adjusted R square	0.29	0.29

Note: *p < 0.1; **p < 0.05; ***p < 0.01

Insurance mode is reference

**Table 4:** Regression results of influence of hospital beds on benefit payments to hospitals

Dependent variable: Payments (US$)		
	Naïve	Robust
Hospital beds	7500*** (5833, 9167)	7500*** (6333, 8833)
Constant	−173.9 E 06*** (−229.3 E 06, −118.4 E 06)	−173.9 E 06*** (−216.2 E 06,-131.6 E 06)
Observations	12	12
R square	0.881	0.881
Adjusted R square	0.869	0.869

Note: *p < 0.1; **p < 0.05; ***p < 0.01

Conversion rate: US$1 = 60

**Table 5:** Difference in claim denials between insurance and trust modes

Dependent variable: CDR. insurance-CDR. trust		
	Naïve	Robust
Private hospitals	−2.43*** (−3.27,-1.59)	−2.43
Constant	2.84*** (2.25, 3.44)	2.84 (−5.34, 11.02)
Observations	12	12
R square	0.76	0.76
Adjusted R square	0.74	0.74

Note: *p < 0.1; **p < 0.05; ***p < 0.01

CDR-Claim Denial Ratio in percent

Reference category is government hospitals

The three most important findings of our study are first the insurance intermediary increases administrative costs; second more beds under the scheme means more expenditure; and third the insurance intermediary hampers payments to government hospitals. We examine the arguments for and against engaging an insurance intermediary.

### Why do we need an insurance intermediary?

There is an impression that an insurer checks the moral hazard. Beneficiary enrolment is not part of RAS because the scheme relies solely on the BPL card issued by government. Absence of enrolment reduces unnecessary administrative cost due to enrolment under the scheme. The mechanism to check moral hazard available to the insurer, which is abuse of benefits by the beneficiary, is preauthorisation. The preauthorisation procedure, laid down by AHCT, is the same for both the implementation modes and the final decision vests with the trust. Besides, it is known that moral hazard is one of the problems faced by health insurers across the world [18, 19]. The belief that insurance intermediary controls moral hazard is untenable. Incidents of unindicated surgeries are detected among treatment procedures implemented under both the modes of RAS [20–22]. SID exists in single-payer government funded schemes as well as in private healthcare insurance settings [23, 24]. Our analysis finds that RAS is functioning as per Roemer's principle independent of the insurance intermediary. Hence, SID cannot be linked to the presence or absence of insurance intermediary. It is seen that more than 30 % of private hospitals under RAS are located in Hyderabad metropolitan region with a population of around 12 % [1]. As per Hart's inverse care law, availability of good medical care tends to vary inversely with the need for it in the population served [25]. This highlights the need for equity in geographical distribution of hospitals.

Patient satisfaction rates above 90 % in any scheme are generally considered to mean good service levels. We find that the patient satisfaction rates during the period when the scheme was entirely administered in trust mode were above 95 % which suggests that even without insurance intermediary the patient satisfaction levels were above the 90 % threshold. This finding about patient satisfaction rates does not support the claim that insurance intermediary is better at handling customer complaints.

RAS engaged the insurance intermediary 22 times during the period 2007–12 and except on two occasions, the hospital claim payments by the insurer did not exceed the premium amount received from AHCT. The cumulative claim ratio of 16.4 % suggests that there is no element of risk absorption by the insurer [5]. Government schemes generally involve large payments unlike the private health insurance schemes. Any miscalculation of bid amount on the part of insurer at the time of bidding is said to lead to disastrous results for the insurer. The IRDA, therefore, does not allow health insurers in India to implement government funded health insurance schemes if a loss is recorded in any government funded scheme during any year. We see an interesting trend in the bidding documents of not only RAS but also other government financed health insurance schemes in India where around 20 % of contract value is allowed to the insurer as administrative cost provided the actual claim payment falls below 80 % [1]. In case the actual claim ratio falls below 80 % a certain amount will be refunded by the insurer to government. Our analysis finds that the average claim ratio under the insurance mode of RAS hovers around 80 %. This suggests that the insurer manages the claims ratio around the level which allows him opportunity of full utilisation of the 20 % administrative cost while at the same time ensuring that no refund is required to be made due to claim ratio falling much below 80 %. The insurer regulates his claims ratio at the desired level based on the principle that addition of beds leads to an additional benefit payments. The insurance intermediary, who is the implementing agency, plays a significant role in adding or removing a hospital under the scheme. Evidence suggests that the insurance intermediary is, therefore, not capable of risk absorption under RAS.

It is generally acknowledged that Government hospitals adopt a negligent attitude in submitting timely claims under RAS. However, such negligence applies equally to the insurance mode as well as trust mode [26]. Assuming that the negligence factor remains the same in both modes, our finding that Government hospitals receive lesser payments from insurance intermediary compared to trust mode negates the claim of better operational efficiency of an insurance intermediary.

The subject of study in this scenario is the difference in payments done under the trust and the insurance modes to public hospitals for the services already rendered by them. Certain patients were treated by the public hospitals under the RAS for which payments had to be made. The fact that some cases were treated under RAS in public hospitals indicates that to the extent of such cases the public hospitals were accessible to the people. Preauthorisation and claims procedures being the same between the trust and insurance modes, ideally there should not be any difference between claims payments under the trust and insurance modes to the public hospitals but our finding was to the contrary.

The state policy of recognising about 87 % of the population to be BPL brings in large numbers under the health insurance net. Such large numbers improve the fund flow forecasts under the scheme. These numbers could be serving as an attraction for the private hospitals to get empanelled under the scheme. The insurance intermediary played an important role in regulating the empanelment of the private hospitals in order to regulate the fund outgo to the hospitals and the trust endorsed this principle.

Administrative costs of private insurance are higher than government financed insurance schemes [27, 28]; our study confirms this finding. Higher administrative costs lead to lower expenditure on patient benefits in view of limited government resources. An insurance intermediary involves higher administrative cost without any real improvement in administrative efficiency, as discussed in the aforementioned sub-section. Therefore engaging additional layers between trust and hospitals while making payments should not be resorted to. RAS was established with the help of an insurance company. It is uncertain as to whether the Trust would have had the capacity and skills to develop the standard operating procedures which now underpin the scheme, without the involvement of the insurance company, especially because the scheme was launched and successfully extended to all parts of this state of approximately 80 million people with rapid speed. The Trust later transferred existing business processes developed by the insurer into their own infrastructure. Other states too, may need such support, and some may continue to need it beyond the set-up phase, depending on their inherent capacity. These business processes are developed by the TPAs on behalf of the insurance companies. Therefore it makes more sense in directly engaging a TPA for establishing the business processes needed for setting up an insurance scheme at a lower cost.

It is argued that the insurance intermediary could have played a role in improving the quality of care in hospitals. The purpose of designing business processes by any private insurer will be to reduce the claim payments to hospitals but not necessarily to improve patient health outcomes. The trust has implemented the business processes under the scheme in consultation with the insurer with the objective of improving patient health outcomes. A separate study is required on whether the business processes under the scheme have led to improved health outcomes.

As discussed, the power of empanelment or removal of hospitals from RAS under the influence of insurer, involves a risk of collusion between the insurance intermediary and the private hospitals resulting in increases in premium charged from government year after year. In Andhra Pradesh scenario where all the private hospitals comprise of two cartels the risk is even higher [29]. The only mechanism which is said to check this risk is the competition among the insurers at the time of bidding. We see not more than one dozen IRDA licenced health insurers in the country, out of which four are large public sector non-life insurers. One can see a risk of cartel formation among the private health insurance firms operating in a country with not more than two dozen big states. It is 'safe' for public-sector insurance firms to lose a contract by quoting a higher bid in any bid seeking to hire an insurance intermediary, rather than bag the contract at a low rate and run a risk of loss. A public-sector insurer is subject to questioning by vigilance and audit agencies if they make a loss. There is a likelihood that the bid amount of public sector insurer is leaked before it files its bid. The prohibition imposed by IRDA on TPAs, who are the business process outsourcing agencies for health insurance companies in India, from serving government sponsored schemes also serves as a disadvantage to the government funded health insurance schemes [30].

All the critical insurance related functions rendered by an insurance company such as enrolment of beneficiaries, collection of premium from beneficiaries, fixation of prices, preauthorisation of treatments and empanelment of hospitals is done by government or trust in the case of RAS. If all that government wants is services offered by a management contractor, engaging an insurance intermediary and transferring tax money in bulk from the state exchequer to an insurer should not be resorted to. Our study finds evidence in support of the tenth recommendation of HLEG that purchases of all health care services under the UHC system should be undertaken directly by government or through quasi-governmental autonomous agencies [4].

States intending to launch a demand side financed scheme would require a state run implementing agency similar to the Trust. During the initial launch and in the later years, RAS experience shows that a private agency is required in order to establish the business processes needed for successful launch of the scheme. The three options currently available in India are the insurance company option, the TPA option and the direct implementation through permanent staff. The first option is the most expensive and the last option is recognised to be the most inefficient in terms of operations. Therefore, we see merit in choosing the option of engaging a TPA for successful roll out of such schemes where cost efficiency is also of paramount importance in spending tax money.

There are limitations to this study. First, a certain portion of administrative cost of the trust such as provision of floor space at subsidised rate to insurer, sharing of a part of the salary bill of field staff and so on might have reduced the administrative cost burden of the insurance intermediary. Though the administrative cost estimates of insurance mode might be underestimated, the inferences drawn remain valid. Second, the assumption that the cost of administering a procedure is independent of district and time period may not be valid. Similarly, all the procedures may not have the same administrative costs. The mix of procedures and districts varied with time under the insurance and the trust modes. At different points of time, all the procedures and all the districts were administered through both the insurance mode and trust mode as well. The standardisation of preauthorisation and claims procedures irrespective of the type of procedure or the district ensures that our assumption does not make our results invalid. Third, towards the end of the study period, starting from August 2011, a policy stipulating that about 192 medical and surgical procedures (reserved procedures) out of the total 938 procedures offered under the scheme could only be performed in public hospitals was introduced. About 192 procedures of low to high value ranging from low to high utilisation were reserved exclusively for public hospitals. Such procedures were being funded through the Trust mode in certain districts and the insurance mode in certain other districts. The only difference is that these procedures are not available in private hospitals. Public hospitals in about half of the districts in the State were receiving funds under the Trust mode while those in the other half were receiving funds through the Insurance mode. This factor could not have influenced the conclusion that CDR for public hospitals in trust mode is significantly lesser than that of the insurance mode because payments to public hospitals were analysed without reference to whether such procedures are available in private hospitals or not. Fourth, this paper does not deal with aspects such as burden of disease or patient attitude towards public and private hospitals. The subject of study of this paper is confined only to cost efficiency of engaging an insurance intermediary. Lastly, the results were based on data pertaining to first six years of RAS implementation which may be considered a very small period. Even though these are initial results, when seen together with the experience of health insurance in various countries from earlier studies our inferences remain valid.

## Conclusion

Rajiv Aarogysri community health insurance scheme is operating on the Roemer principle and therefore suffers from supplier induced demand, irrespective of whether or not an insurance intermediary is engaged. Engaging an insurance intermediary not only reduces benefit payments for beneficiaries but also reduces fund flow to government hospitals. Aarogyasri Health Care Trust itself being the single-payer health insurance agency of government, adding an additional layer in the form of an insurance intermediary between the trust and the hospitals should not be done. We therefore, conclude with the very important policy recommendation that proposals for engaging insurance intermediaries for administering government funded health insurance schemes should be abandoned and instead business process outsourcing agencies should be engaged in their place directly.

## Abbreviations

AHCT: Aarogyasri Health Care Trust
BCR: Benefit Cost Ratio
BPL: Below Poverty Line
CDR: Claim Denial Ratio
GoAP: Government of Andhra Pradesh
HLEG: High Level Expert Group
IRDA: Insurance Regulatory and Development Authority of India
IT: Information Technology
RAS: Rajiv Aarogyasri Scheme
SID: Supplier Induced Demand
TPA: Third Party Administrator
UHC: Universal Health Coverage
